# Response regulator VemR regulates the transcription of flagellar rod gene *flgG* by interacting with σ^54^ factor RpoN2 in *Xanthomonas citri *ssp. *citri*

**DOI:** 10.1111/mpp.12762

**Published:** 2018-11-28

**Authors:** Wei Wu, Zhiwen Zhao, Xuming Luo, Xiaojing Fan, Tao Zhuo, Xun Hu, Jun Liu, Huasong Zou

**Affiliations:** ^1^ State Key Laboratory of Ecological Pest Control for Fujian and Taiwan Crops, Fujian University Key Laboratory for Plant–Microbe Interaction, College of Plant Protection Fujian Agriculture and Forestry University Fuzhou 350002 China; ^2^ State Key Laboratory of Plant Genomics, Institute of Microbiology Chinese Academy of Sciences Beijing 100101 China

**Keywords:** cell motility, regulation, RpoN2, VemR, *Xanthomonas citri *ssp. *citri*

## Abstract

*Xanthomonas citri *ssp. *citri*, a polar flagellated bacterium, causes citrus canker disease worldwide. In this study, we found that the *X. citri *ssp. *citri* response regulator VemR plays a regulatory role in flagellum‐derived cell motility. Deletion of the *vemR* gene resulted in a reduction in cell motility, as well as reductions in virulence and exopolysaccharide production. Reverse transcription‐polymerase chain reaction (RT‐PCR) demonstrated that *vemR* is transcribed in an operon together with *rpoN2* and *fleQ.* In the *vemR* mutant, the flagellar distal rod gene *flgG* was significantly down‐regulated. Because *flgG* is also *rpoN2* dependent, we speculated that VemR and RpoN2 physically interact, which was confirmed by yeast two‐hybrid and maltose‐binding protein (MBP) pull‐down assays. This suggested that the transcription of *flgG* is synergistically controlled by VemR and RpoN2. To confirm this, we constructed a *vemR* and *rpoN2* double mutant. In this mutant, the reductions in cell motility and *flgG* transcription were unable to be restored by the expression of either *vemR* or *rpoN2* alone. In contrast, the expression of both *vemR* and *rpoN2 *together in the double mutant restored the wild‐type phenotype. Together, our data demonstrate that the response regulator VemR functions as an RpoN2 cognate activator to positively regulate the transcription of the rod gene *flgG *in *X. citri *ssp. *citri.*

## Introduction

*Xanthomonas citri *ssp. *citri* (*Xcc*) is the causal agent of citrus canker disease, and its single polar flagellum plays multiple roles during infection. Flagellar motility governs the translational movement towards favourable environments in response to physical or chemical attractants (Ottamann and Miller, 1997). Moreover, flagellar‐dependent cell motility is essential for the establishment of a mature biofilm, and is thus involved in the ability to attach to host surfaces and to elicit canker lesions on citrus (Li and Wang, 2011; Malamud *et al*., 2011; Viducic *et al*., 2017; Yaryura *et al*., 2015). In addition, the filament flagellin has been shown to be a pathogen‐associated molecular pattern, inducing innate immune responses in host plants (Zipfel *et al*., 2004). Flagellin‐induced innate immunity plays a critical role in determining the resistance of citrus species to *Xcc *(Shi *et al*., 2015, 2016)*.*

The bacterial flagellum extends from the cell surface to form a helical propeller. This pronounced structure is composed of three evidently contiguous substructures: the basal body, hook and helical filament (Snyder *et al*., 2009). The basal body spans the bacterial cell envelope and comprises a ‘drive‐shaft’ rod with a series of rings. The rod is responsible for the stable, high‐speed rotation of the motor driving filament rotation (Minamino and Namba, 2004). The assembly of the proximal rod requires cooperative interactions between the FlgB, FlgC and FlgF proteins, whilst FlgG forms the most distal part (Homma *et al*., 1990). FlgG tends to form β‐amyloid‐like fibrils through interactions with its unfolded regions under most crystallization conditions (Saijo‐Hamano *et al*., 2004). In *Rhodobacter sphaeroides*, the* flgGHIJKL* genes are expressed as a single transcriptional unit, and their transcription is dependent on the σ^54^ factor, encoded by the *rpoN* gene (González‐Pedrajo *et al*., 2002). Although the composition of this operon varies among bacterial strains, its dependence on σ^54^ appears to be conserved. For example, *flgG*, *flhB* and* fliC* are significantly down‐regulated in *rpoN2* mutants of *Xanthomonas campestris* pv. *campestris *(Hu *et al*., 2005).

Many polar flagellated bacteria require the σ^54^ factor to activate the transcription of genes essential for flagellar biosynthesis. The σ^54^ factor and RNA polymerase form a transcriptionally inactive closed complex consisting of holoenzyme bound to double‐stranded DNA with the consensus promoter sequence YTGGCACGrNNNTTGCW (Barrios *et al*., 1999). This sequence is recognized by the σ^54^ factor and usually centred around nucleotide positions –12 and –24 from the transcription start (Morett and Buck, 1989). To initiate transcription, the closed complex must interact with a transcriptional activator, involving nucleotide hydrolysis (Studholme and Dixon, 2003). The activation of σ^54^‐dependent transcription is highly regulated by environmental stimulation through the regulatory modules of transcriptional activators (Shingler, 2011; Siegel and Wemmer, 2016). The sensory modules of transcriptional activators are often present in the N‐terminal region and include CheY‐like response regulator domains, PAS domains, GAF domains, PRD modules and V4R domains (Shingler, 2011).

In *Xanthomonas*, the *vemR* gene encodes an atypical response regulator. It contains CheY‐like receiver, but lacks an output domain (Qian *et al*, 2008). Deletion of *vemR* in *X. campestris* pv. *campestris *leads to reductions in virulence, exopolysaccharide (EPS) production and motility (Tao and He, 2010). The function of response regulators is routinely controlled by phosphorylation, which is executed by cognate histidine kinases (Galperin, 2006). However, mutation of the putative phosphorylation sites in VemR does not significantly affect EPS synthesis, motility or virulence (Tao and He, 2010). This suggests that VemR may function through an alternative pathway to modulate bacterial phenotypes. In *Xcc*, there are two copies of *rpoN*, known as *rpoN1* and *rpoN2*, the latter of which is reported to be more closely related to the transcription of flagellar biosynthesis genes. In a previous study, we inactivated *rpoN1* and *rpoN2 *in *Xcc *and found that swimming motility and the expression of flagellar biosynthesis genes were reduced more significantly in the *rpoN2* mutant (Gicharu *et al*., 2016). The *rpoN2* and *vemR* genes are adjacent in the *Xcc* genome. Thus, in this study, we aimed to determine whether these genes are involved in an associated pathway to modulate cell motility.

## Results

### Deletion of *vemR* leads to reduced virulence on citrus and a hypersensitive response (HR) on non‐host tomato

**Figure 1:**
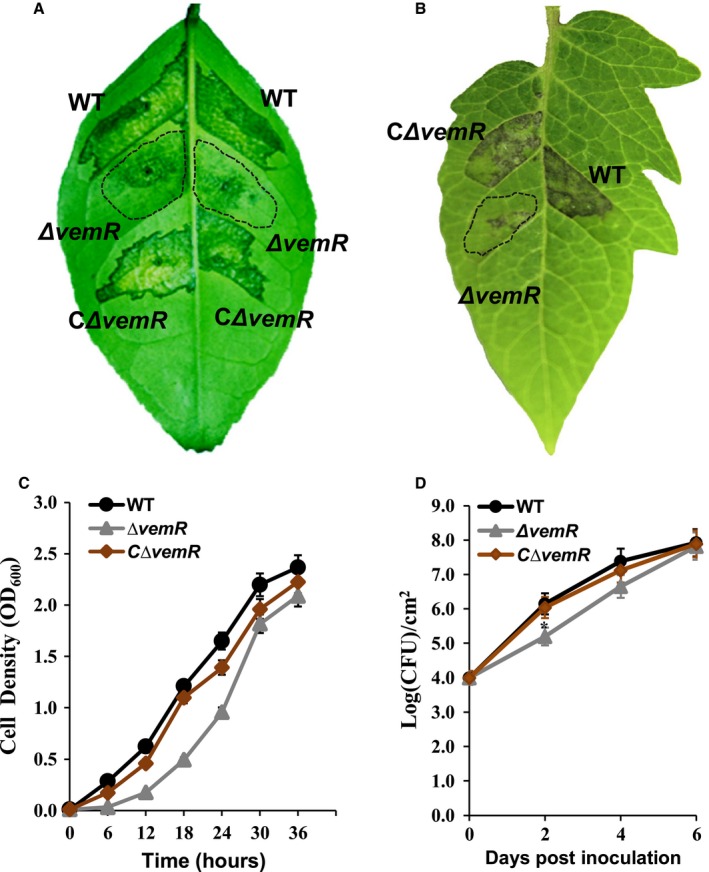
Phenotypes of *vemR* mutants*. *(A) Citrus canker symptoms on *Citrus paradisi *Macf. cv. Duncan. Disease symptoms were scored every day and photographed at 7 days post‐inoculation (dpi). (B) Hypersensitive response (HR) on *Solanum lycopersicum *at 2 dpi. The *Xanthomonas citri* ssp. *citri *cells [10^7^ colony‐forming units (CFU)/mL] were inoculated into citrus and tomato plants for pathogenicity and HR assays, respectively. The inoculation areas of the *vemR* mutants are indicated by the broken lines. (C) Bacterial growth in nutrient‐rich nutrient broth (NB) liquid medium. Each strain was prepared at an initial concentration of OD_600_ (optical density at 600 nm) = 0.01, and bacterial multiplication was measured from the OD_600_ value every 6 h. The values shown are the means of three technical repeats with standard deviations. (D) Bacterial growth in citrus plants. Bacteria were recovered from leaves inoculated with *X. citri* ssp. *citri *cells at a concentration of 10^8^ CFU/mL every 2 days after inoculation. Values shown are the means of three technical repeats with standard deviations. WT, wild‐type.

To investigate *vemR* function, a non‐polar deletion mutant of *Xcc *29‐1 was established, and the deletion was confirmed by polymerase chain reaction (PCR) (Fig. S1, see Supporting Information). Compared with wild‐type *Xcc* 29‐1, the *vemR* mutant exhibited a marked reduction in virulence on a citrus host at 7 days post‐inoculation (dpi) (Fig. 1A). At 2 dpi of tomato leaves, the *vemR* mutant exhibited reduced ability to induce an HR (Fig. 1B). The plasmid pBB‐vemR, carrying the *vemR* gene under the control of the *XAC1347* promoter, was used for complementation. Both virulence and the HR reaction were restored by the introduction of the complementary recombinant pBB‐vemR into the mutant (Fig. 1A,B).

Mutagenesis of *vemR* minimally affects bacterial growth. Bacterial growth was evaluated at 6‐h intervals in nutrient broth (NB) liquid medium from an original cell density of OD_600_ (optical density at 600 nm) = 0.01. At each testing time point, the cell density of the *vemR* mutant was slightly lower than that of the wild‐type. At 36 h post‐inoculation (hpi), the cell density of the *vemR* mutant reached OD_600_ = 2.0 (Fig. 1C). To evaluate bacterial growth in host plants, cell numbers were measured every 2 days after the inoculation of citrus leaves. At 2 dpi, the cell density of wild‐type *Xcc* 29‐1 reached 10^6^ colony‐forming units (CFU)/cm^2^, exhibiting a high proliferation rate in host plants. It reached 10^7^ CFU/cm^2^ at 4 dpi and 10^8^ CFU/cm^2^ at 6 dpi. In contrast, the number of *vemR* mutant cells was ~10^5^ CFU/cm^2 ^at 2 dpi, whereas, at 6 dpi, the mutant cell number was not distinctly different from that of the wild‐type (Fig. 1D).

### *fleQ, vemR* and *rpoN2* are expressed as a single transcriptional operon

**Figure 2:**
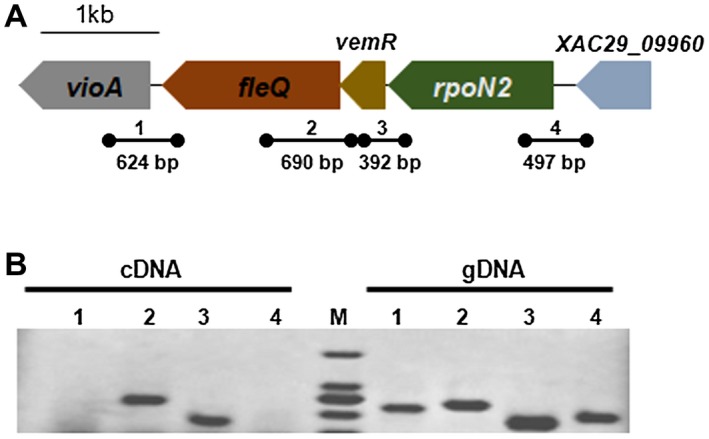
Schematic representation of the *fleQ*‐*vemR*‐*rpoN2* operon and the reverse transcription‐polymerase chain reaction (RT‐PCR) strategy. (A) Arrows with different colours depict the open reading frames of the operon and their lengths in base pairs. The threads represent the sizes and approximate locations in the PCR analysis with primer sets. (B) PCR products using cDNA and gDNA as templates. 1, 2, 3 and 4 represent the products of the corresponding junction fragments in (A). The DNA marker was DL2000.

In the genome of *Xcc* 29‐1 (GenBank accession no. NC_020800.1), the *vemR* gene is annotated as a response regulator encoded by *XAC29_09950*. A σ^54^ factor gene, *rpoN2*, is found downstream of *vemR,* and the *fleQ* gene is found upstream (Fig. 2A). To determine whether *fleQ*, *vemR *and *rpoN2 *are expressed as a single operon, four specific primer sets were designed to amplify junction fragments of 624, 690, 392 and 497 bp (Fig. 2A). The primers were used for PCR following the synthesis of first‐strand cDNA as a template. As shown in Fig. 2B, the 392‐bp fragment covering *rpoN2* and *vemR*, as well as the 690‐bp fragment covering *vemR* and *fleQ*, were successfully amplified from the reverse transcript cDNA. In contrast, the primer set covering *fleQ* and *vioA* did not produce the 624‐bp fragment, and the primer set covering *rpoN2* and its downstream gene was unable to amplify the 497‐bp fragment (Fig. 2B). All primer sets were effective at amplifying the desired DNA fragments from gDNA (Fig. 2B). This indicates that *rpoN2*, *vemR* and *fleQ* are expressed as a single transcriptional operon.

### *vemR* regulates bacterial swimming by modulation of *flgG* transcription

**Figure 3:**
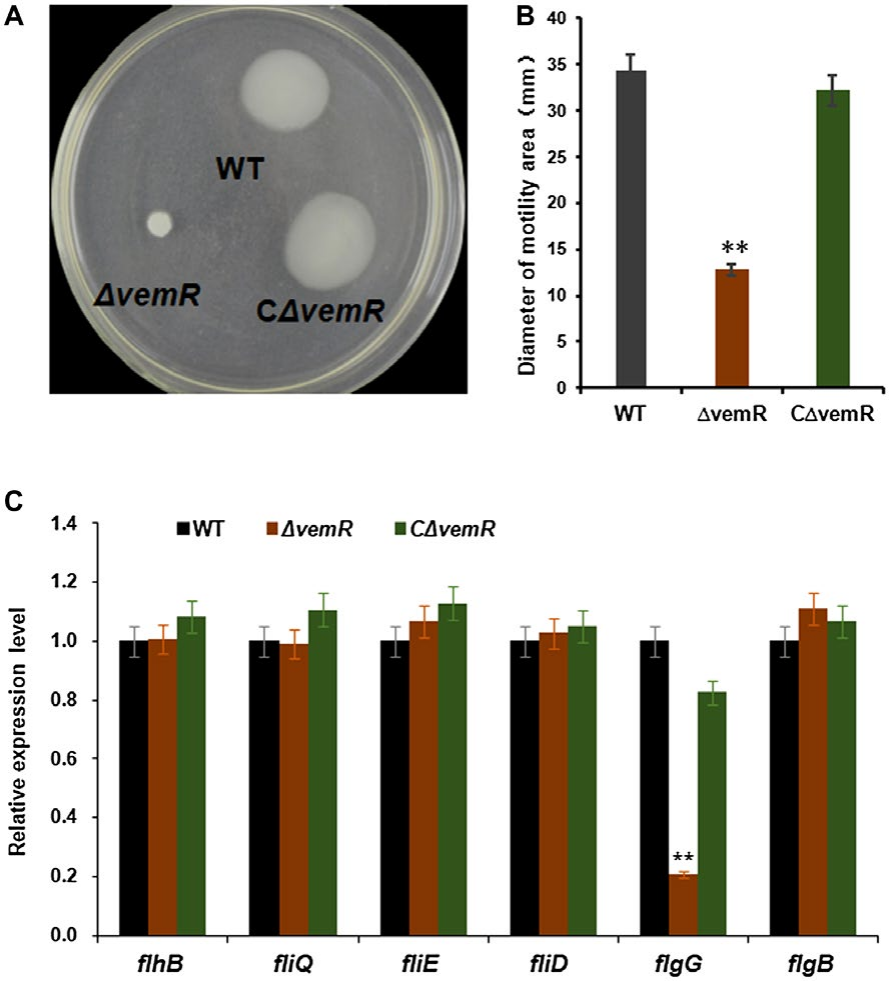
Swimming motility of the *vemR* mutant and relative expression levels of eight flagellar biosynthesis genes. (A) Swimming motility of the *vemR* mutant on a semisolid plate. Cell suspensions (2 µL) were spotted onto 0.3% agar MMX plates, and bacterial swimming was determined from the diameter of each colony at 3 days post‐inoculation. (B) Measurement of colony diameters. The data were derived from triple repeats. The asterisks in the horizontal data column indicate significant differences in colony diameter at *P* = 0.01 according to Student’s *t*‐tests. (C) Quantitative reverse transcription‐polymerase chain reaction (qRT‐PCR) analysis of the expression of eight flagellar biosynthesis genes. Total RNA was extracted from *Xanthomonas citri *ssp.* citri* cells cultured in MMX liquid medium. For each gene, the expression level in the wild‐type (WT) was calculated as ‘1’ using *gyrA* as an internal control. Statistical analysis was conducted using Student’s *t*‐tests. **P ˂ 0.01 vs. WT.

The swimming ability of *Xcc* strains was assayed on low‐agar MMX plates. After incubation at 28 °C for 3 days, the *vemR* mutant exhibited a considerably reduced motility compared with that of wild‐type *Xcc* 29‐1. The wild‐type colony diameter reached an average of 34.4 mm, whereas the *vemR* mutant colony diameter was 12.8 mm (*P* < 0.01), representing a 62.8% reduction in the mutant (Fig. 3A). Wild‐type swimming motility was restored in the complemented strain (Fig. 3A), indicating that flagellar‐dependent swimming ability was impaired in the *vemR* mutant. Our previous study demonstrated that the expression levels of eight flagellar biosynthesis genes (*flhF*, *flhB*, *fliQ*, *fliL*, *fliE*, *fliD*, *flgG* and *flgB*) were down‐regulated in the *rpoN2* mutant (Gicharu *et al*., 2016). In the *vemR* mutant, the transcription levels of *flhF*, *flhB*, *fliQ*, *fliL*, *fliE*, *fliD *and *flgB* were not significantly different from wild‐type levels (Fig. 3B). In contrast, the transcription of *flgG* was reduced by about 80% compared with that of the wild‐type (*P* < 0.01). The specific expression pattern of *flgG* suggests that it is co‐regulated by *rpoN2* and *vemR* in* Xcc*.

### VemR physically interacts with σ^54^ factor RpoN2

**Figure 4:**
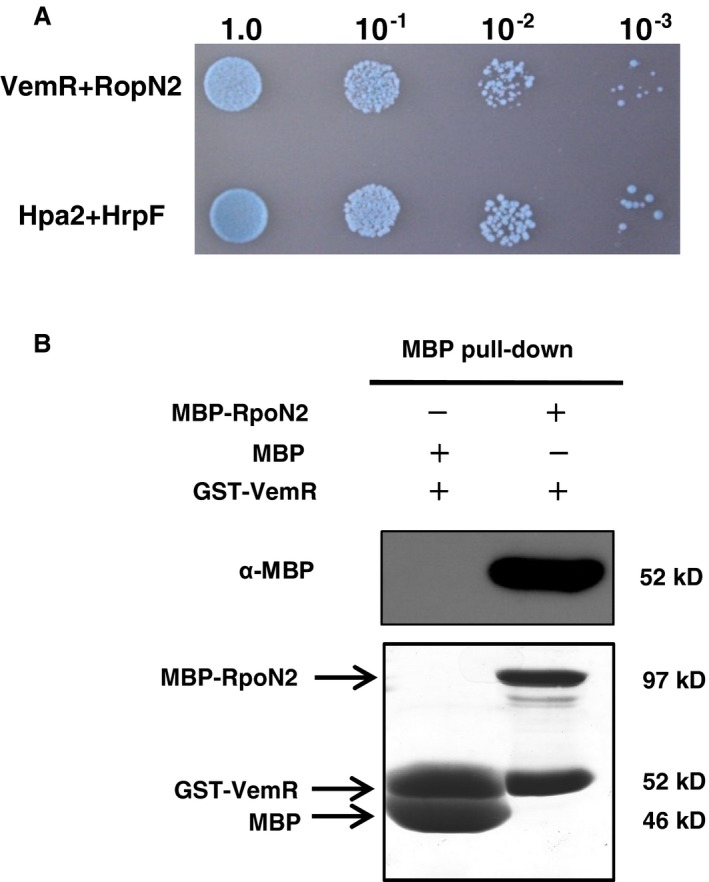
VemR physically interacts with RpoN2. (A) Yeast two‐hybrid assays showing the interaction between VemR and RpoN2. The positive transformants were prepared to a cell density of OD_600_ (optical density at 600 nm) = 1.0 and diluted to a 10‐fold series. For each concentration series, 2‐μL suspensions were spotted and incubated on synthetic defined SD/‐Ade/‐Leu/‐Trp/‐His plates supplied with 20 μg/mL X‐α‐galactosidase (X‐α‐gal) for 4 days. The interaction between Hpa2 and HrpF was used as a positive control. (B) VemR interacts with RpoN1 *in vitro* according to maltose‐binding protein (MBP) pull‐down assays. MBP‐RpoN2 (3 µg) and GST‐VemR were incubated overnight at 4 °C with 300 μL of amylose resin. After the eluted protein samples had been boiled, samples were separated on a 12% sodium dodecylsulfate‐polyacrylamide gel electrophoresis (SDS‐PAGE) gel and immunoblotted with anti‐MBP. The negative control included the incubation of 3 µg maltose binding protein (MBP) protein with GST‐VemR.

Because the *flgG* promoter contains a consensus sequence recognized by σ^54^, the regulatory role of VemR in *flgG* transcription prompted us to ask whether VemR and RpoN2 could physically interact at the protein level. To test this hypothesis, VemR and RpoN2 were cloned into pGADT7 and pGBKT7, respectively. A yeast two‐hybrid assay indicated VemR and RpoN2 interaction activity (Fig. 4A). To confirm the interaction observed in yeast, maltose‐binding protein (MBP) pull‐down assays were carried out using GST‐VemR as bait. Consistent with the two‐hybrid result, pull‐down assays revealed that VemR interacts with the RpoN2 protein (Fig. 4B).

### Transcription of *flgG* requires both *vemR* and *rpoN2*

**Figure 5:**
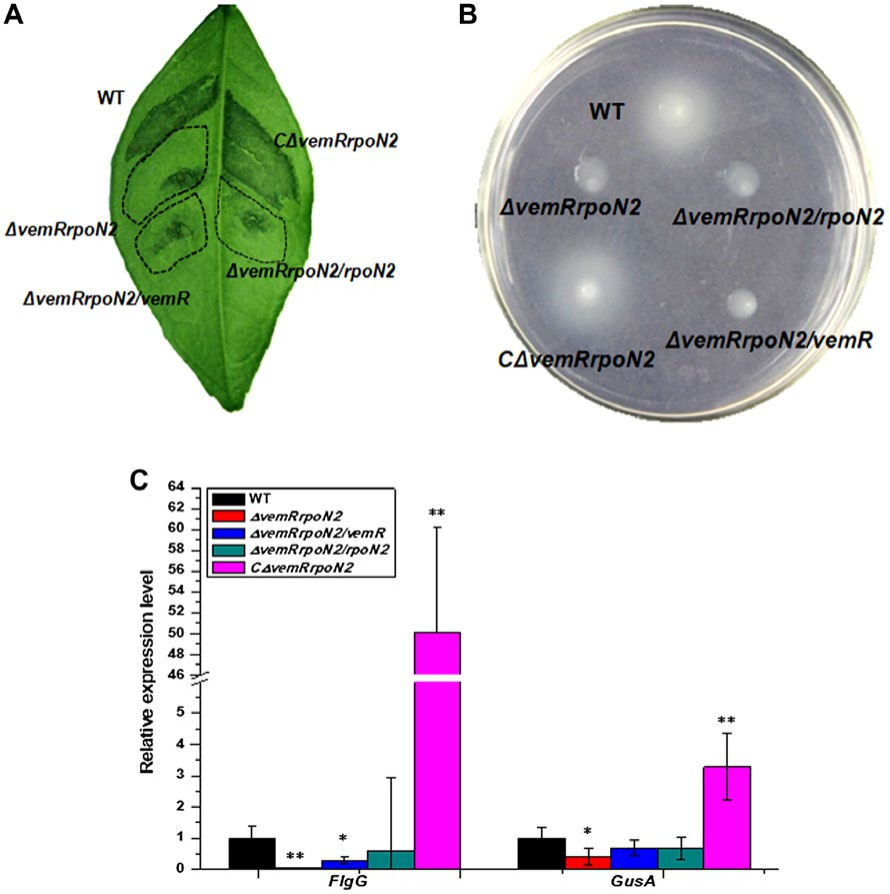
VemR and RpoN2 are both required for *flgG* transcription. (A) Phenotype of *vemR* and *rpoN2* double mutant in citrus plants. Bacterial suspensions of 10^7^ colony‐forming units (CFU)/mL were inoculated onto citrus using an infiltration method. Disease symptoms were photographed at 7 days post‐inoculation. The weak canker symptoms caused by the mutants are highlighted by the broken lines. (B) Swimming motility of *vemR* and *rpoN2* double mutant on low‐agar plates. Cell suspensions (2 µL) were spotted onto 0.3% agar MMX plates, and photographs were taken at 3 days post‐inoculation. (C) Transcription of* flgG* and promoter‐monitored *GusA *gene in the double mutant. Total RNA was extracted from cells cultured in MMX liquid medium. The expression of *flgG* or *GusA* in the wild‐type (WT) was set as ‘1’. Statistical analysis was conducted using Student’s *t*‐tests. *P ˂ 0.05, **P ˂ 0.01 vs. WT.

To determine whether the transcription of *flgG* requires the presence of both *vemR* and *rpoN2*, we constructed a *vemR* and *rpoN2* double mutant (Fig. S2, see Supporting Information). The double mutant exhibited phenotype alterations similar to those of the *vemR* mutant, with reduced cell swimming motility on soft agar plates and reduced virulence in citrus plants (Fig. 5A,B). Wild‐type phenotypes were restored by complementation with pBB‐vemRrpoN2 expressing both *vemR* and *rpoN2*. In contrast, expression of either *vemR* or *rpoN2* alone could not restore wild‐type swimming motility and virulence phenotypes (Fig. 5A,B). Accordingly, the transcription of *flgG* was restored in the double mutant by simultaneous expression of *vemR* and *rpoN2*, whereas transcription was not restored by the expression of either gene individually (Fig. 5C). To confirm this, the *flgG* promoter was cloned into pGD960 and fused with the *GusA* reporter gene. The expression of *GusA* under the *flgG* promoter showed similar patterns to *flgG* expression. Namely, the expression of *GusA* was markedly reduced in the double mutant and was not restored by the expression of either *vemR* or *rpoN2 *alone (Fig. 5C).

## Discussion

The response regulator VemR is essential for the full virulence of *Xcc*. Based on the available genome sequences, the *vemR* gene has been identified in several plant‐pathogenic bacteria, including *X. campestri*s pv. *campestris* (Qian *et al*., 2005; da Silva *et al*., 2002), *X. oryzae *pv. *oryzae* (Salzberg *et al*., 2008) and *Xcc *(da Silva *et al*., 2002). Its function has been elucidated previously in *X. campestris *pv. *campestris*. Deletion of this gene results in reductions in motility, virulence and EPS production (Tao and He, 2010). In the present study, the deletion of *vemR* in *Xcc* 29‐1 resulted in similar phenotypic alterations. In addition to reduced virulence and HR induction, the EPS yield of the *vemR* mutant was half that of the wild‐type *Xcc* 29‐1, representing a significant decrease (Fig. S3, see Supporting Information).

Based on RT‐PCR experiments,* fleQ*,* vemR* and *rpoN2 *were shown to be expressed in a single operon in *Xcc*. Similar results have been reported from *X. campestri*s pv. *campestris *and *X. oryzae *pv. *oryzae *(Tao and He, 2010; Tian *et al*., 2015)*. *This conserved genetic organization suggests that the *fleQ‐vemR‐rpoN2* operon may possess a similar biological function in all plant‐pathogenic members of *Xanthomonas*. However, the contributions of the three genes to cell motility and virulence differ among pathogens. For instance, mutation of *vemR* does not lead to cell motility impairments in *X. campestris* pv. *campestris* ATCC 33913 or *X. oryzae* pv. *oryzae* PXO99A (Qian *et al*., 2008; Tian *et al*., 2015). In addition, deletion of *fleQ* impairs swimming motility in *X. oryzae* pv. *oryzae *PXO99A and *X. campestris* pv. *campestris* Xc 17, but has no effect in *X. campestris* pv. *campestris* ATCC 33913 (Hu *et al*., 2005; Qian *et al*., 2008; Tian *et al*., 2015).

In a typical two‐component signal transduction system, the N‐terminal receiver domain of the response regulator accepts a phosphoryl group from a histidine kinase, and the phosphorylated response regulator activates its C‐terminal output domain to trigger an adaptive response by the modulation of gene expression or the cellular machinery (Galperin, 2006). VemR was first reported in *X. campestris* pv. *campestris* based on the complete genome sequence, and it was found to promote bacterial survival under osmolarity stress, sodium challenge, heat shock and exposure to sodium dodecylsulfate (Qian *et al*., 2008). Although the VemR protein harbours a CheY‐like receiver, it possesses no output domains (Qian *et al*., 2005). Moreover, mutation of the putative phosphorylation sites does not significantly reduce *X. campestris* pv. *campestris* EPS synthesis, motility or virulence (Tao and He, 2010). This suggests that this protein may execute its function via protein–protein interactions. In this study, we found that VemR regulates the transcription of the flagellar rod gene *flgG* by interacting with the σ^54^ factor, RpoN2. We speculate that the virulence reduction in the *vemR* mutant may be a result of the reduced flagellar‐derived cell motility in this mutant (Malamud *et al*., 2011). The mechanism by which it regulates EPS production, however, requires further study.

Our understanding of RpoN as a regulator of gene expression is largely derived from studies demonstrating its requirement for the expression of the glutamine synthetase gene *glnA* in *Escherichia coli* (Hunt and Magasanik, 1985; Pahel and Tyler, 1979) and the nitrogen fixation genes in *Rhizobium *(Somerville and Kahn, 1983). A number of soil‐borne and plant‐associated bacteria possess two genes encoding RpoN proteins (Gicharu *et al*., 2016; Kullik *et al*., 1991; Ray *et al*., 2015; Tian *et al*., 2015). Although the two genes exhibit high levels of identity, the two proteins are not interchangeable (Lundgren *et al*., 2015; Michiels *et al*., 1998). In contrast with the *rpoN1* mutant strain, inactivation of the *rpoN2 *gene in *Rhizobium etli* did not produce any phenotypic defects during free‐living growth. However, symbiotic nitrogen fixation was reduced by approximately 90% in the *rpoN2* mutant, whereas the nitrogen fixation of the *rpoN1* mutant was the same as that of the wild‐type (Michiels *et al*., 1998). In plant‐associated *Xanthomonas*, *rpoN2* plays a more important role in flagellar‐derived cell motility and virulence. The *rpoN2* mutant of *X. oryzae* pv. *oryzae* exhibits a loss of swimming motility and significant down‐regulation of *fliA*, *flgM*, *flgG*, *flhB* and *fliC* (Tian *et al*., 2015). Our previous study demonstrated that both *rpoN1* and *rpoN2* play regulatory roles in swimming motility, but that *rpoN2* has a dominant role in this regulation (Gicharu *et al*., 2016).

**Figure 6:**
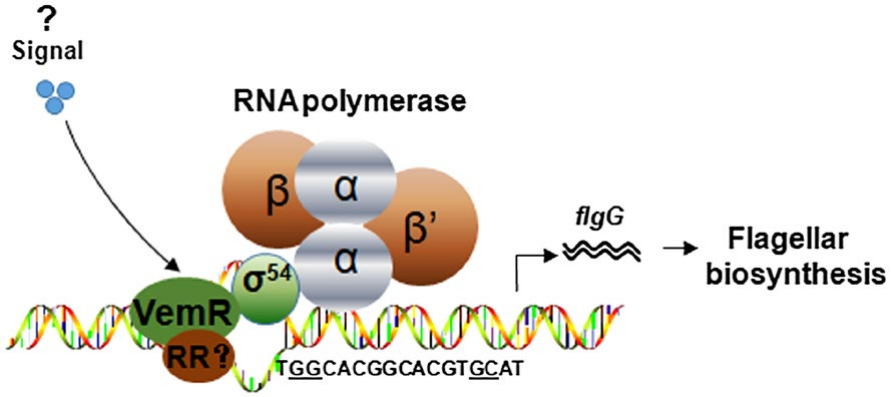
Proposed model for transcription of* flgG *synergistically controlled by VemR and RpoN2 in *Xanthomonas citri *ssp. *citri*. VemR contains a CheY‐like receiver domain that responds to environmental stimuli. As VemR does not have an output domain, a typical response regulator (RR) or transcriptional activator is probably recruited to interact with VemR. RpoN2 is responsible for recognizing the –24/–12 consensus sequence (TGGCACGGCACGTGCAT) in the *flgG* promoter. Using this protein–protein interaction module, the transcription of *flgG* is initiated by RNA polymerase, and the resulting protein plays a role in flagellar biosynthesis.

RpoN proteins rely on the C‐terminus to bind with specific promoter sites and form an RpoN–RNAP holoenzyme complex by interacting with RNA polymerase for transcription initiation (Hong *et al*., 2009). RpoN–RNAP is unable to spontaneously isomerize from a closed complex to a transcriptionally competent open complex (Xu and Hoover, 2001). As an additional step before transcription initiation, the closed complex interacts with a transcriptional activator through the N‐terminus (Glyde *et al*., 2017). These transcriptional activators usually bind at least 100 bp upstream of the promoter site, and DNA looping is required for the activator to contact the closed complex and catalyse the formation of the open promoter complex (Morett and Segovia, 1993). In this scenario, the target gene promoter contains a transcriptional activator binding site outside of the consensus DNA sequence recognized by RpoN at the −24/−12 sites (Barrios *et al*., 1999). We assessed the expression of eight σ^54^‐dependent flagellar biosynthesis genes in the *vemR* mutant and found that the transcription of the flagellar rod gene *flgG* was markedly down‐regulated. Therefore, our data suggest a model whereby VemR regulates flagellar‐derived cell motility by interacting with RpoN2, rather than receiving a phosphorylation signal from a histidine kinase sensor (Fig. 6). Furthermore, a response regulator is likely to be recruited for this regulation, as VemR does not contain an output domain. Future studies should therefore aim to characterize this putative response regulator.

In conclusion, our results indicate that the response regulator VemR controls the transcription of the flagellar rod gene* flgG* by interacting with RpoN2. These findings provide a better understanding and new perspective on the biological functions of response regulators without classical phosphorylation sites and output domains. Although the regulatory mechanism of VemR and its effects on virulence and EPS production remain to be determined, future studies may provide a rational basis for controlling citrus canker disease using VemR as a therapeutic target.

## Experimental Procedures

### Bacterial strains, plasmids and culture conditions

The bacterial strains and plasmids used in this study are listed in Table S1 (see Supporting Information). The* Xcc *strains were cultivated in NB medium or NB containing 1.5% agar (NA) at 28 °C (Ye *et al*., 2013). *Escherichia* *coli* strains were routinely cultured in Luria–Bertani (LB) medium at 37 °C. In yeast hybridization experiments, yeast strain AH109 was grown in yeast extract peptone dextrose (YPD) medium at 30 °C. Antibiotics were applied at the following concentrations: ampicillin (Ap), 100 μg/mL; kanamycin (Km), 50 μg/mL; gentamycin (Gm), 10 μg/mL.

### Generation of non‐polar deletion mutant

The non‐polar deletion mutant *ΔvemR *was constructed according to the method described previously (Zhuo *et al*., 2015). In brief, the primer pairs vemR1.F/vemR1.R and vemR2.F/vemR2.R were used to amplify the flanking DNA fragments. The two flanking sequences were fused with overlapping PCR and then inserted into the suicide vector pKMS1 to generate pKMS‐vemR for mutant isolation (Table S1). A counter‐selectable marker (*SacB*) enables the pKMS1 vector to be used as a tool for non‐marker mutagenesis in *Xcc* (Zou *et al*., 2011). After selection on NA plates supplemented with 10% sucrose, the primer set vemR1.F/vem2R was used to identify the desired *ΔvemR* deletion mutant*.*

The same genetic manipulation methods were used to produce a *vemR* and *rpoN2* double mutant. The primers used for the construction of the mutants are listed in Table S2 (see Supporting Information). The DNA fragments flanking the target gene sequences were amplified to generate pKMS‐vemRrpoN2. Following transformation into wild‐type* Xcc* 29‐1, the deletion mutant *ΔvemRΔrpoN2* was obtained.

### Construction of complementary plasmids

In order to constitutively express the target genes, the promoter region of the *XAC1347* gene was first cloned into pBBR1MCS‐5 at the *Kpn*I and *Xho*I sites (Kovach *et al*., 1994), generating pBB‐P1347 (Table S1). The primer pair CvemR.F and CvemR.R was used to amplify the full‐length open reading frame of *vemR* (Table S2). The resulting PCR product was then inserted into pBB‐P1347 at the *Eco*RI and *Xba*I sites, generating pBB‐vemR. The *rpoN2* gene was cloned into pBB‐P1347 at the *Xho*I and *Eco*RI sites, generating pBB‐rpoN2. The *rpoN2* gene was additionally cloned into pBB‐vemR to generate pBB‐vemRrpoN2 (Table S1). The complementary plasmids were introduced into the corresponding mutants to investigate phenotype restoration.

### Pathogenicity and HR assays

The cultured *Xcc* mutant strains were centrifuged at 3500 ***g*** for 10 min and suspended in sterile distilled water to a final concentration of 10^7^ CFU/mL (OD_600_ = 0.03). Bacterial suspensions were injected into grapefruit (*Citrus paradisi *Macf. cv. Duncan) leaves with a needleless syringe. Disease symptoms were scored and photographed at 7 dpi. For analysis of the HR, bacterial suspensions of 10^7^ CFU/mL were inoculated into tomato (*Solanum lycopersicum *L*. *cv*. *Zhongshu No. 4) leaves. Plant reactions were viewed at 2 dpi. The tests were repeated at least three times.

### Assays for bacterial growth in NB medium and *in planta*

The *Xcc* strains were cultured in NB medium until they reached an OD_600_ = 1.0. Then, cells were subcultured (1 : 100) in fresh NB and incubated for another 16 h until they reached an OD_600_ = 0.6. After centrifugation at 6000 g for 10 min at 4 °C, the cell pellets were resuspended in sterilized water to an OD_600_ = 1.0. The cell suspension was subcultured (1 : 100) in NB liquid medium, and growth was measured by determination of the OD_600_ value every 6 h. All experiments were repeated three times.

For growth assays *in planta*, cultured cells were adjusted to a final concentration of 10^8^ CFU/mL (OD_600_ = 0.3). After inoculation of citrus leaves, leaf samples of 0.8 cm^2^ were collected to calculate the cell numbers every 2 days after inoculation. The bacterial number approximately represented the CFU/cm^2^ of the leaf area, and the standard deviation was calculated using colony counts from three triplicate spots for each of the three samples per time point per inoculum. The experiments were repeated three times.

### Motility assay

Motility was assessed on 0.3% agar MMX plates as described previously (Zhuo *et al*., 2015). *Xcc* 29‐1 and the derived mutants were grown in NB medium and suspended in MMX liquid medium at a concentration of OD_600_ = 1.0 after centrifugation. Then, 2 μL of each cell sample was inoculated onto the surfaces of semisolid plates. Photographs were taken after bacterial growth for 3 days. The diameters of the swimming zones around each inoculation spot indicated movement ability. The experiments were repeated at least three times.

### Semi‐quantitative and real‐time RT‐PCR

To study the full‐length mRNA of the operon, specific primers (Table S2) were designed to amplify the junction regions (Fig. 1). Total RNA was extracted from *Xcc* cells cultured in MMX liquid medium with an RNA Prep Pure Cell/Bacteria Kit (Tiangen Biotech, Beijing, China). The quality of total RNA was analysed by gel electrophoresis, and the quantity was measured by a spectrophotometer. To remove contaminating genomic DNA, a PrimeScript RT Reagent Kit with gDNA Eraser (TaKaRa Bio, Dalian, China) was used before reverse transcription. Then, 2 μg of total RNA was reverse transcribed into single‐stranded cDNA using AMV reverse transcriptase (TaKaRa Bio). The primers used for RT‐PCR analysis are listed in Table S2. Those used for the *flgG* gene, as well as the seven other flagellar biosynthesis genes, have been validated previously (Gicharu *et al*., 2016). The expression of *gyrA* was used as an internal control.

In the semi‐quantitative RT‐PCR for operon identification, PCR thermal cycling conditions consisted of initial denaturation at 94 °C for 5 min, followed by 32 cycles of DNA denaturation at 94 °C for 30 s, primer annealing at 52 °C for 40 s and primer extension at 72 °C for 1 min, with a final elongation step at 72 °C for 10 min. For quantitative evaluation of gene expression levels, real‐time RT‐PCR was conducted using SYBR Premix Ex Taq (TaKaRa Bio). The PCR parameters were as follows: denaturation at 95 °C for 30 s and 40 cycles of 95 °C for 5 s and 58 °C for 30 s.

### Yeast two‐hybrid assay

The yeast two‐hybrid assay was performed according to the manufacturer’s instructions (Clontech, Fremont, CA, USA). VemR was cloned into the pGADT7‐AD vector, and RopN2 was cloned into the pGBKT7‐BD vector. The two resulting constructs were then co‐transformed into the yeast strain AH109. The positive transformants were screened on SD/‐Ade/‐Trp/‐His and SD/‐Ade/‐Leu/‐Trp/‐His. Subsequently, the interaction between VemR and RopN2 was confirmed by incubation on synthetic defined SD/‐Ade/‐Leu/‐Trp/‐His plates supplied with 20 μg/mL X‐α‐galactosidase (X‐α‐gal). The cultured transformants were prepared to a cell density of OD_600 _= 1.0, and then diluted to a 10‐fold series (Liu *et al*., 2009). For each concentration series, 2‐μL suspensions were spotted onto plates and incubated for 4 days. pAHpa2 and pBHrpF were used as positive controls in transformation isolation, bacterial growth and galactosidase assays during all the yeast two‐hybrid experiments (Li *et al*., 2011).

### MBP pull‐down assay

The full‐length coding region of *vemR* was cloned into pET41a(+) at the *Bam*HI and *Eco*RI sites, and that of *rpoN2 *was cloned into pMAl‐C4X at the *Bam*HI and *Xho*I sites. The two constructs were transformed into BL21(DE3) cells for induction with 1.0 mm isopropyl‐β‐d‐thiogalactopyranoside (IPTG). Bacterial cells were harvested and resuspended in 10 mm phosphate‐buffered saline. Following sonication, insoluble cell debris was removed by centrifugation. GST‐VemR proteins were immobilized on a glutathione resin according to the manufacturer’s instructions (Genescript, Nanjing, China). MBP‐RpoN2 was purified by amylose affinity chromatography. The MBP pull‐down assay was performed using the method described previously (Liu *et al*. 2011). In brief, 3 µg of both MBP‐RpoN2 and GST‐VemR were incubated overnight at 4 °C with 300 μL of amylose resin (Sigma, Shanghai, China). The beads were collected by centrifugation and washed with 0.1% Triton X‐100 and increasing concentrations of NaCl to eliminate spurious protein interactions. Proteins were eluted from the amylose resin, boiled in 1 × SDS loading buffer and electrophoresed on a 12% sodium dodecylsulfate‐polyacrylamide gel electrophoresis (SDS‐PAGE) gel before being immunoblotted with anti‐MBP. A negative control was performed by incubation of 3 µg maltose binding protein (MBP) protein with GST‐VemR.

## Supporting information


**Fig. S1** ****Molecular analysis of the *ΔvemR *mutant of *Xanthomonas citri* ssp.* citri. *Differences in the sizes of the polymerase chain reaction (PCR) products from wild‐type *Xcc *29‐1 and *ΔvemR *were revealed using the primers vemR1.F and vemR2.R. The PCR product from the mutant was smaller than that of the wild‐type as a result of deletion of the *vemR* coding sequence. Lane M, DNA marker DL5000; lane 1, wild‐type; lane 2, *vemR* mutant.

**Fig. S2** ****Molecular identification of the *rpoN2 *and *vemR* double mutant*.* A DNA fragment was amplified from the double mutant using the primers DM6869.1.F and DM6869.2.R. In the wild‐type, the polymerase chain reaction (PCR) product was 3185 bp, whereas that of the double mutant was 1385 bp. The PCR product from the wild‐type is indicated with an arrow. Lane M, DNA marker DL5000; lane 1, wild‐type; lane 2, double mutant.

**Fig. S3** ****Exopolysaccharide (EPS) products in the *ΔvemR *mutant. *Xanthomonas citri *ssp. *citri* (*Xcc*) was grown in 100 mL of nutrient broth (NB) medium at 28 °C with constant shaking at 200 rpm for 3 days. EPS was precipitated from the culture supernatant by the addition of 300 mL of ethanol. After drying to a constant weight at 55 °C, the precipitate was weighed. All experiments were repeated at least three times.

**Table S1** ****Bacterial strains and plasmids used in this study.

**Table S2** ****Primers used in this study.
