# Supplementary figures and images for: Response regulator VemR regulates the transcription of flagellar rod gene flgG by interacting with σ54 factor RpoN2 in Xanthomonas citri ssp. citri
Source: Mol Plant Pathol. 2018 Nov 28;20(3):372–81. doi: 10.1111/mpp.12762 (PMC6637908; doi:10.1111/mpp.12762)

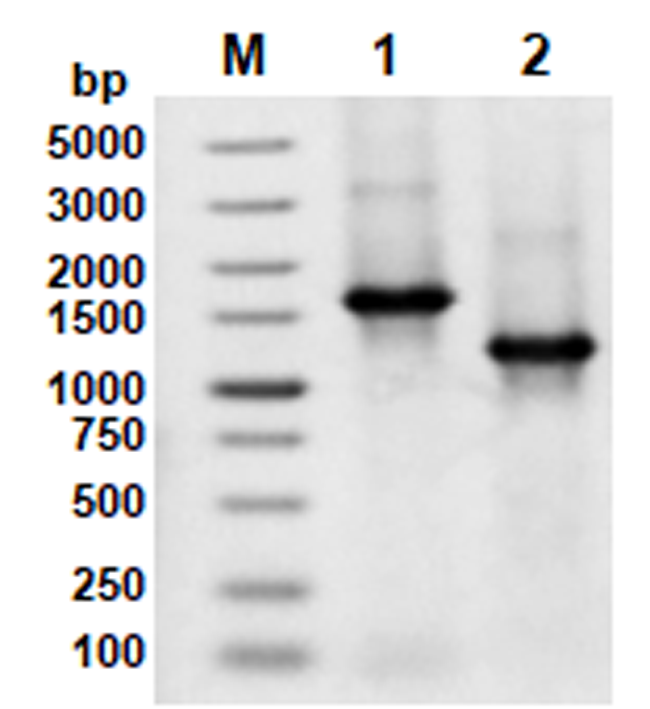

Supplement: Supplementary file 1 — Fig. S1 Molecular analysis of the ΔvemR mutant of Xanthomonas citri ssp. citri. Differences in the sizes of the polymerase chain reaction (PCR) products from wild‐type Xcc 29‐1 and ΔvemR were revealed using the primers vemR1.F and vemR2.R. The PCR product from the mutant was smaller than that of the wild‐type as a result of deletion of the vemR coding sequence. Lane M, DNA marker DL5000; lane 1, wild‐type; lane 2, vemR mutant. [file MPP-20-372-s001.tif]

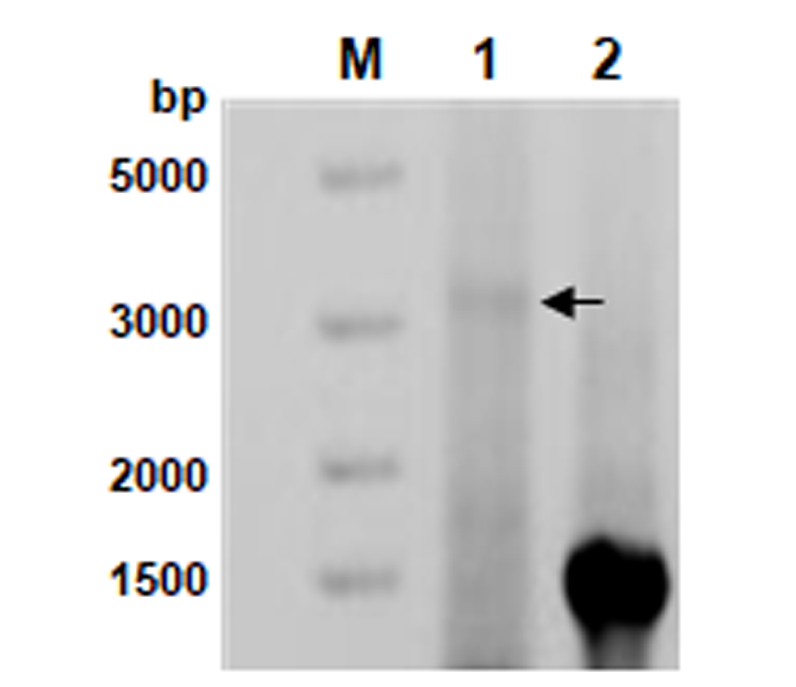

Supplement: Supplementary file 2 — Fig. S2 Molecular identification of the rpoN2 and vemR double mutant. A DNA fragment was amplified from the double mutant using the primers DM6869.1.F and DM6869.2.R. In the wild‐type, the polymerase chain reaction (PCR) product was 3185 bp, whereas that of the double mutant was 1385 bp. The PCR product from the wild‐type is indicated with an arrow. Lane M, DNA marker DL5000; lane 1, wild‐type; lane 2, double mutant. [file MPP-20-372-s002.tif]

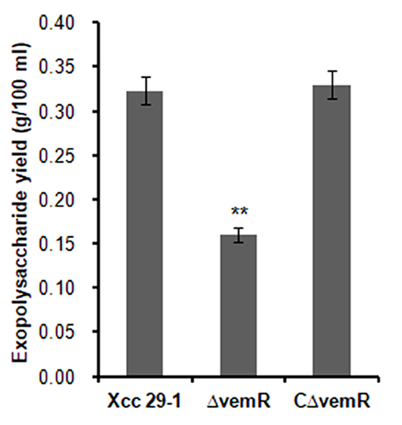

Supplement: Supplementary file 3 — Fig. S3 Exopolysaccharide (EPS) products in the ΔvemR mutant. Xanthomonas citri ssp. citri (Xcc) was grown in 100 mL of nutrient broth (NB) medium at 28 °C with constant shaking at 200 rpm for 3 days. EPS was precipitated from the culture supernatant by the addition of 300 mL of ethanol. After drying to a constant weight at 55 °C, the precipitate was weighed. All experiments were repeated at least three times. [file MPP-20-372-s003.tif]
