# Supplementary material for: Response regulator VemR regulates the transcription of flagellar rod gene flgG by interacting with σ54 factor RpoN2 in Xanthomonas citri ssp. citri
Source: Mol Plant Pathol. 2018 Nov 28;20(3):372–81. doi: 10.1111/mpp.12762 (PMC6637908; doi:10.1111/mpp.12762)
Supplement: Supplementary file 4 — Table S1 Bacterial strains and plasmids used in this study. [file MPP-20-372-s004.docx]

Table S1. Bacterial strains and plasmids used in this study

| **Strain or plasmid** | **Relevant characteristics** | **Reference** |
| --- | --- | --- |
| **Strains** | | |
| *Xanthomonas citri* subsp. *citri* | | |
| *Xcc* 29-1 | Wild-type strain isolated from *Citrus sinensis* in Jiangxi Province, China | Ye *et al*., 2013 |
| *ΔvemR* | A non-polar deletion mutant of *vemR* gene derived from *Xcc* 29-1 | This study |
| *CΔvemR* | Gm^r^, *ΔvemR* carrying the complementation construct pBB-*vemR* | This study |
| *ΔvemRrpoN2* | A non-polar mutant of *vemR* and *rpoN2* double mutant derived from *Xcc* 29-1 | This study |
| *ΔvemRrpoN2/vemR* | Gm^r^, *ΔvemRrpoN2* double mutant complemented by pBB-*vemR* | This study |
| *ΔvemRrpoN2/rpoN2* | Gm^r^, *ΔvemRrpoN2* double mutant complemented by pBB-*rpoN2* | This study |
| *CΔvemRrpoN2* | Gm^r^, *ΔvemRrpoN2* double mutant complemented by pBB-*vemRrpoN2* | This study |
| *Xcc* 29-1(pRG960-P*_flgG_*) | Sp^r^, *Xcc* 29-1 carrying pRG960-P*_flgG_* | This study |
| *ΔvemRrpoN2*(pRG960-P*_flgG_*) | Sp^r^, *ΔvemRrpoN2* carrying pRG960-P*_flgG_* | This study |
| *ΔvemRrpoN2/vemR*(pRG960-P*_flgG_*) | Sp^r^, *ΔvemRrpoN2/vemR* carrying pRG960-P*_flgG_* | This study |
| *ΔvemRrpoN2/rpoN2*(pRG960-P*_flgG_*) | Sp^r^, *ΔvemRrpoN2/rpoN2* carrying pRG960-P*_flgG_* | This study |
| *CΔvemRrpoN2*(pRG960-P*_flgG_*) | Sp^r^, *CΔvemRrpoN2* carrying pRG960-P*_flgG_* | This study |
| *Escherichia coli* | | |
| DH5α | *F^-^ recA hsdR17 (rk^−^, mk^+^) ϕ80lacZ∆M15* | Clontech |
| BL21(DE3) | *F^-^, ompT, hsdSB (rB^-^mB^-^), gal, dcm* | Novagen |
| Yeast | | |
| AH109 | MATa, *trp1-901, leu2-3, 112, ura3-52, His3-200, gal4, gal80, LYS2::GAL1UAS-GAL1TATA-His3* | Clontech |
| **Plasmids** | | |
| pKMS1 | Km^r^, suicide vector derived from pK18mobGII, *sacB*^+^ | Zou *et al*., 2011 |
| pKMS-vemR | Km^r^, a 1191-bp fusion cloned in pKMS1 with a 404-bp deletion comprising 384 bp of *vemR* coding sequence and 20-bp sequence upstream of translation start codon | This study |
| pKMS-vemRrpoN2 | Km^r^, a 1385-bp fusion cloned in pKMS1 with a 1864-bp deletion comprising *vemR* and *rpoN2* coding sequence as well as 68-bp sequence upstream of *rpoN2* translation start codon | This study |
| pBBR1MCS-5 | Gm^r^, 4.7-kb broad-host range plasmid, *lacZ* | Kovach *et al*., 1994 |
| pBB-PXAC1347 | Gm^r^, pBBR1MCS-5 harboring a 553-bp DNA fragment of *XAC1347* gene promoter | This study |
| pBB-vemR | Gm^r^, the *vemR* gene cloned in pBB-PXAC1347 under control of *XAC1347* promoter | This study |
| pBB-rpoN2 | Gm^r^, the *rpoN2* gene cloned in pBB-PXAC1347under control of *XAC1347* promoter | This study |
| pBB-vemRrpoN2 | Gm^r^, the *vemR* and *rpoN2* genes cloned in pBB-PXAC1347 under control of *XAC1347* promoter | This study |
| pGADT-7 | Amp^r^, *GAL4*(768-881) AD, *LEU2*, HA epitope tag | Clontech |
| pGADT7-VemR | Amp^r^, the 384-bp full-length *vemR* gene cloned in pGADT7-AD at *Nde*I and *Eco*RI sites | This study |
| pGBKT-7 | Km^r^, *GAL4*(1-147) BD, *TRP1*, c-myc epitope tag, | Clontech |
| pGBKT7-RpoN2 | Km^r^, the 1404-bp full-length *rpoN2* gene cloned in pGBKT7-BD at *Nde*I and *Eco*RI sites | This study |
| pET41a (+) | Km^r^, IPTG-inducible expression vector | Novagen |
| pET41-VemR | Km^r^, a 384-bp *vemR* gene in pET41a(+) for expressing VemR-GST fusion | This study |
| pMAL-4X-1 | Amp^r^, an expressing vector with a maltose binding protein (MBP) tag | New England Biolabs |
| pMAL-4X-1-RpoN2 | Amp^r^,a 1404-bp *rpoN2* gene in pMAL-4X-1 for expressing RpoN2-MBP fusion | This study |
| pRG960 | Sp^r^, broad-host-range vector carrying a promoterless *gusA* gene with start codon | Van den Eede *et al*. 1992 |
| pRG960-P*_flgG_* | Sp^r^, a 170-bp *flgG* gene promoter region ligated into pRG960 | This study |

**REFERENCES**

**Kovach, M. E., Phillips, R. W., Elzer, P.H., Roop, R. and Peterson, K. M.** (1994) pBBR1MCS: a broad-host-range cloning vector. *Gene* **166,** 175–176.

**Van den Eede, G., Deblaere, R., Goethals, K., Van Montagu, M. and Holsters, M.** (1992) Broad host range and promoter selection vectors for bacteria that interact with plants. *Mol. Plant-Microbe Interact.* **5,** 228–234.

**Ye, G., Hong, N., Zou, L.-F., Zou, H.-S., Zakria, M., Wang, G.-P., and Chen, G.-Y**. (2013) *Tale-*Based genetic diversity of Chinese isolates of the citrus canker pathogen *Xanthomonas citri* subsp. *citri*. *Plant Dis.* **97,** 1187–1194.

**Zou, L., Li, Y. and Chen, G.** (2011) A non-marker mutagenesis strategy to generate poly-hrp gene mutants in the rice pathogen Xanthomonas oryzaepv. oryzicola. Agri. Sci. China **10,** 1139*–*1150.
