# Supplementary material for: Response regulator VemR regulates the transcription of flagellar rod gene flgG by interacting with σ54 factor RpoN2 in Xanthomonas citri ssp. citri
Source: Mol Plant Pathol. 2018 Nov 28;20(3):372–81. doi: 10.1111/mpp.12762 (PMC6637908; doi:10.1111/mpp.12762)
Supplement: Supplementary file 5 — Table S2 Primers used in this study. [file MPP-20-372-s005.docx]

Table S2. Primers used in this study

| **Primer pair** | **Sequence(5’-3’)** | **Cutting sites** | **Description or purpose** |
| --- | --- | --- | --- |
| **Primers used for molecular cloning** | | | |
| vemR1.F/vemR1.R | TCGGATCCTGTCGATGCGCTATGAGA  GGCAAGCAGCGGTTTAGT | *Bam*HI-*Pst*I | A 440-bp DNA fragment upstream of *vemR* gene |
| vemR2.F/vemR2.R | ACTAAACCGCTGCTTGCCGTCCCGCATTCTGTTGATC  TCCTGCAGCACGCGCAACAACTT |  | A 751-bp DNA fragment downstream of *vemR* gene |
| PXAC1347.F/PXAC1347.R | TCGGTACCGGCAATCACCGTGCTAAT  TCCTCGAGTTTCAATCCTCGTCGTTAGA | *Kpn*I-*Xho*I | A 553-bp DNA fragment of *XAC1347* gene promoter sequence |
| CvemR.F/CvemR.R | TCGAATTCATGAGCAAACTCACCGTGCTG  TCTCTAGATCACTCATTCCTGGCTCCTTC | *Eco*RI-*Xba*I | A 384-bp DNA fragment of *vemR* gene |
| CrpoN2.F/CrpoN2.R | TCCTCGAGATGAAGACGACCATTTCCG  TCGAATTCTTATCCTGCCCGGGCAAGC | *Xho*I- *Eco*RI | A 1404-bp DNA fragment of *rpoN2* gene |
| vemR-AD.F/vemR-AD.R | TCCATATGAGCAAACTCACCGTG  TCGAATTCTCACTCATTCCTGGCTCCTT | *Nde*I-*Eco*RI | A 384-bp full length of *vemR* gene fused in pGADT7 vector |
| rpoN2-BD.F/rpoN2-BD.R | TCCATATGAAGACGACCATTTCCGCCCAGC  TCGAATTCTTATCCTGCCCGGGCAAGCAGCGGT | *Nde*I-*Eco*RI | A 1404-bp full length of *rpoN2* gene fused in pGBKT7 Vector |
| vemR-GST.F/vemR-GST.R | TCGGATCCAGCAAACTCACCGTG  TCGAATTCTCACTCATTCCTGGCTCCTT | *Bam*HI-*Eco*RI | A 381-bp DNA fragment of *vemR* inserted into pET41a(+) for fused with GST-tag |
| rpoN2-MBP.F/rpoN2-MBP.R | GGATTTCAGAATTCGGATCCATGAAGACGACCATTTCCGC  AAGCTTGCCTGCAGCTCGAGTTATCCTGCCCGGGCAAGCA | *Bam*HI-*Xho*I | A 1404-bp DNA fragment inserted into pMAL-c4X a for expressing MBP-tagged RpoN2 |
| DM6869.1.F/DM6869.1.R | TCGGATCCTGGTGCTGATGGACCTGTC  TCTCTAGATTCTAATCAGGGAGTCGGGT | *Bam*HI-*Xba*I | A 634-bp DNA fragment upstream of *rpoN2* gene |
| DM6869.2.F/DM6869.2.R | TCTCTAGAGTCCCGCATTCTGTTGAT  TCCTGCAGCACGCGCAACAACTT | *Xba*I- *Pst*I | A 751-bp DNA fragment downstream of *vemR* gene (Same as vemR2.F/vemR2.R) |
| 81P.F/81P.R | TCCTGCAGTCGACAGACCCAGCTTTTG  CCGGATCCTGACCCGATTCCTCGTTAGAC | *Pst*I*- Bam*HI | A 170-bp *flgG* promoter region cloned in pRG960 vector |
| **Primers used for operon characterization** | | | |
| 7069.F/7069.R | TGGCCGCCAAACATCACAA; ACGTCCCACATCGTGCTTTC | | A 497-bp DNA fragment between *XAC1970* and *rpoN2* gene |
| 6968.F/6968.R | CCGCACCACTAAACCGCTG; ATTGGCATGACTTCCTGGATATAG | | A 392-bp DNA fragment between *rpoN2* gene and *vemR* gene |
| 6867.F/6867.R | TGTCAGCAAGCTGTCCTATATCCA; ACAATTCGCTTTCCAGCAGATC | | A 690-bp DNA fragment between *vemR* gene and *fleQ* gene |
| 6766.F/6667.R | TTGGTGGAGAAGCTGCGCA; TGAGGTTGTAGATGTCGGCGA | | A 624-bp DNA fragment between *fleQ* gene and *vioA* gene |
| *gyrA*.F/*gyrA*.R | CTGGACCAACACTGACACTGAG; GTCTCCTTAGAGTTCCCACCAT | | a 423-bp *gyrA* gene |
| **Primers used for qRT-PCR analysis** | | | |
| *flhF* | CTGCTGTCCAAGCGTCTGCC; TTGGCGATGGTGGTGGTCTT | | 107-bp |
| *flhB* | GCTGATGAACCCGACCCACT; GAAGGCCATTTCGTCCACCC | | 100-bp |
| *fliQ* | TGACTGAATTGCGTGGTGGC; GGTTCGTTCAACTGGGTGGC | | 121-bp |
| *fliL* | CACCTGCTGATGCTGCTGTC; GCAATTCTTCGACGCACTTC | | 142-bp |
| *fliE* | ACCGAGCTTCAGCGAGACCT; GACCATGACCCGTGCCAGAT | | 127-bp |
| *fliD* | TCGGTGGAAGTGGTCTCGTT; GTCGGTGCCGCTGATGTCTA | | 138-bp |
| *flgG* | CAACAACCTGGCCAATACCAATAC; GGTACAACAGGTCTTCGAACGC | | 72-bp |
| *flgB* | GACCACCGACGCCAAGCATT; ATCGGGATCGACGGTATTGC | | 112-bp |
| *GusA* | TAGAAACCCCAACCCGTGAA; TTGCCCGGCTTTCTTGTAAC | | 120-bp |
| *gyrA* | TGGCCTCAAGCCTGTGCACCGG; GACGATACGCGCCGACTTGAAG | | 100-bp |

The 5′ end of each primer contains a restriction enzyme site for cloning into the expression plasmids
